# Ocular Bacterial Infections: A Ten-Year Survey and Review of Causative Organisms Based on the Oklahoma Experience

**DOI:** 10.3390/microorganisms11071802

**Published:** 2023-07-13

**Authors:** Roger A. Astley, Md Huzzatul Mursalin, Phillip S. Coburn, Erin T. Livingston, James W. Nightengale, Eddy Bagaruka, Jonathan J. Hunt, Michelle C. Callegan

**Affiliations:** 1 Department of Ophthalmology, University of Oklahoma Health Sciences Center, Oklahoma City, OK 73104, USA; roger-astley@ouhsc.edu (R.A.A.); mdhuzzatul-mursalin@ouhsc.edu (M.H.M.); phillip-coburn@ouhsc.edu (P.S.C.); nightengalewil@gmail.com (J.W.N.); 2 Department of Microbiology and Immunology, University of Oklahoma Health Sciences Center, Oklahoma City, OK 73104, USA; erin.arroz@gmail.com; 3 Department of Biology, Oklahoma Christian University, Edmond, OK 73013, USA; e.bagaruka@eagles.oc.edu (E.B.); jonathan.hunt@oc.edu (J.J.H.); 4 Dean McGee Eye Institute, Oklahoma City, OK 73104, USA

**Keywords:** ocular infection, keratitis, conjunctivitis, endophthalmitis, bacteria, *Staphylococcus*, survey

## Abstract

Ocular infections can be medical emergencies that result in permanent visual impairment or blindness and loss of quality of life. Bacteria are a major cause of ocular infections. Effective treatment of ocular infections requires knowledge of which bacteria are the likely cause of the infection. This survey of ocular bacterial isolates and review of ocular pathogens is based on a survey of a collection of isolates banked over a ten-year span at the Dean McGee Eye Institute in Oklahoma. These findings illustrate the diversity of bacteria isolated from the eye, ranging from common species to rare and unique species. At all sampled sites, staphylococci were the predominant bacteria isolated. Pseudomonads were the most common Gram-negative bacterial isolate, except in vitreous, where *Serratia* was the most common Gram-negative bacterial isolate. Here, we discuss the range of ocular infections that these species have been documented to cause and treatment options for these infections. Although a highly diverse spectrum of species has been isolated from the eye, the majority of infections are caused by Gram-positive species, and in most infections, empiric treatments are effective.

## 1. Introduction

The eye is arguably the most vulnerable organ in the human body. Despite being exposed to organisms, allergens, and physical insults from the external environment, the eye must maintain a healthy and transparent surface in order to allow the retina to be exposed to light for proper vision. The eye must also refrain from responding to these threats as other organs do because those responses can be damaging to the eye’s purpose of sensing light. The surface of the eye is also host to a distinct microbiome which serves to control pathogen growth and provides protection without instigating unnecessary inflammation. Under normal physiological conditions, ocular barriers such as the lids, tear film, and antimicrobial factors physically protect the eye, and the innate and adaptive immune systems, along with the microbiome, prevent the growth of harmful organisms [1,2]. When these protections fail due to systemic illness, physical insult, contact lens wear, or various environmental insults, infection may result [1,2,3]. That said, the normal flora is the predominant source of pathogens that cause bacterial conjunctivitis [4,5], keratitis [6], and postoperative endophthalmitis [7,8].

When an ocular infection does occur, prompt and effective treatment is necessary to prevent damage from both the infection and the immune response. Clinical decisions affecting the management of ocular infections are based on the identification of the pathogen. When culture results return, the rational question is, “is this bacterium a pathogen or a commensal?” Some species of bacteria are always viewed as pathogens, but many ocular bacteria can be pathogenic or commensal depending on the ocular conditions. That is where the science and art of determining an empirical treatment intersect. Knowledge of normal and pathogenic ocular bacteria is vital for prompt and effective treatment [7,9]. This paper reviews common, uncommon, and very rare ocular bacterial isolates and their pathogenic potential and reports comparisons with species included in a collection of ocular isolates banked over a ten-year span.

**Figure 1:**
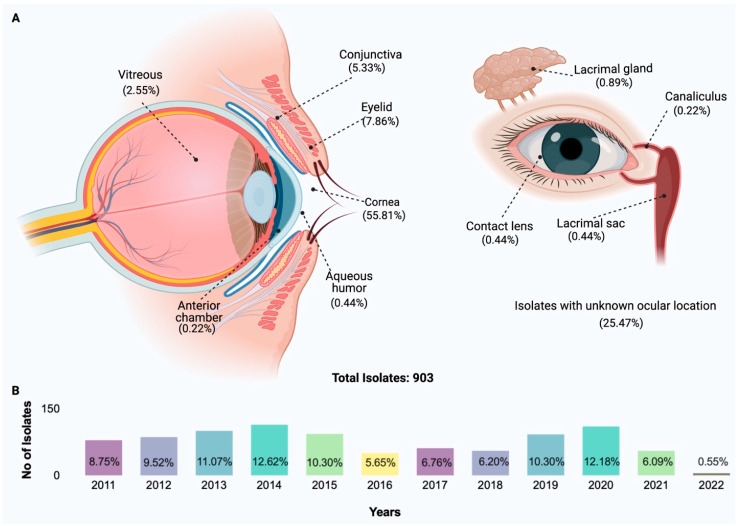
Distribution of ocular bacterial isolates collected according to anatomic site (**A**), and distribution of the number of ocular bacterial isolates collected over the ten-year period of March 2011 to March 2022 (**B**).

**Figure 2:**
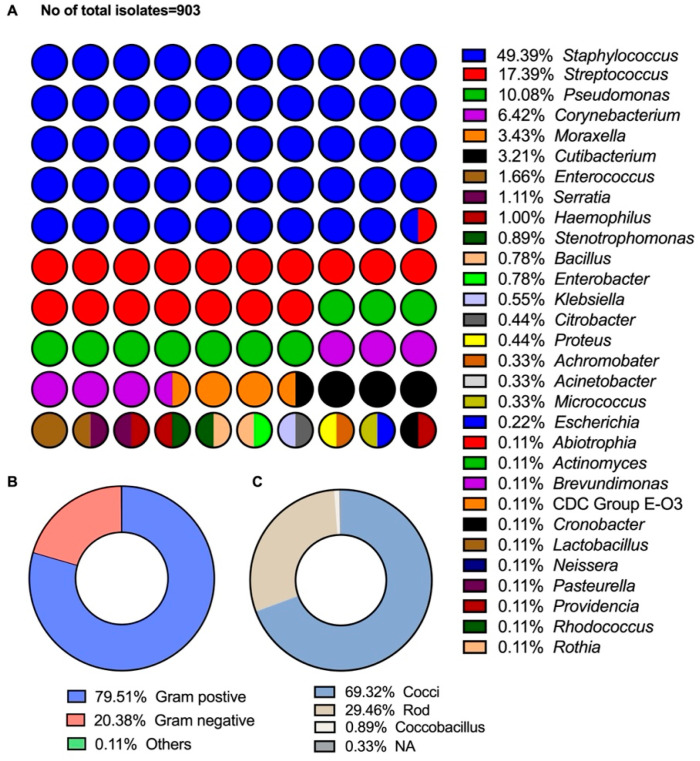
Distribution of bacteria, staining reaction, and morphology. Data are represented as the percentage of bacterial genera (**A**), Gram stain reaction (**B**), and bacterial morphology (**C**) of isolates in the ocular bacterial isolate survey.

The data presented below were compiled from a survey of a collection of bacterial species in the Ocular Isolate Bank located at the Dean McGee Eye Institute in Oklahoma City, Oklahoma. Collected isolates from ocular bacterial infections were dated from March 2011 to March 2022. Clinical data were not available in the information provided for the survey, but anatomic location information was available for 71.9% of isolates. Original bacterial identification was performed offsite using a Bruker MALDI-Biotyper and confirmed when necessary in-house using routine microbiological methods. Polymerase chain reaction (PCR) analysis of staphylococcal virulence factors was conducted as previously described [10]. Figure 1 depicts the anatomical diversity of isolates in the collection. Of the isolates with a designated anatomical location, 55.81% were from the cornea, 7.86%from the eyelid, 5.33%from the conjunctiva, 2.55%from the vitreous, 0.44% from the aqueous humor, and 0.22% from the anterior chamber. Other anatomical locations included the lacrimal gland (0.89%) and sac (0.44%), canaliculus (0.22%), and from contact lens (0.44%). Of the 903 isolates, 79.51% were Gram-positive, and 20.38% were Gram-negative (Figure 2). Of the Gram-positive isolates, *Staphylococcus* and *Streptococcus* were the most commonly isolated genera (49.39% and 17.39%, respectively). *Pseudomonas* was the most frequently isolated Gram-negative bacteria (10.08%).

**Figure 3:**
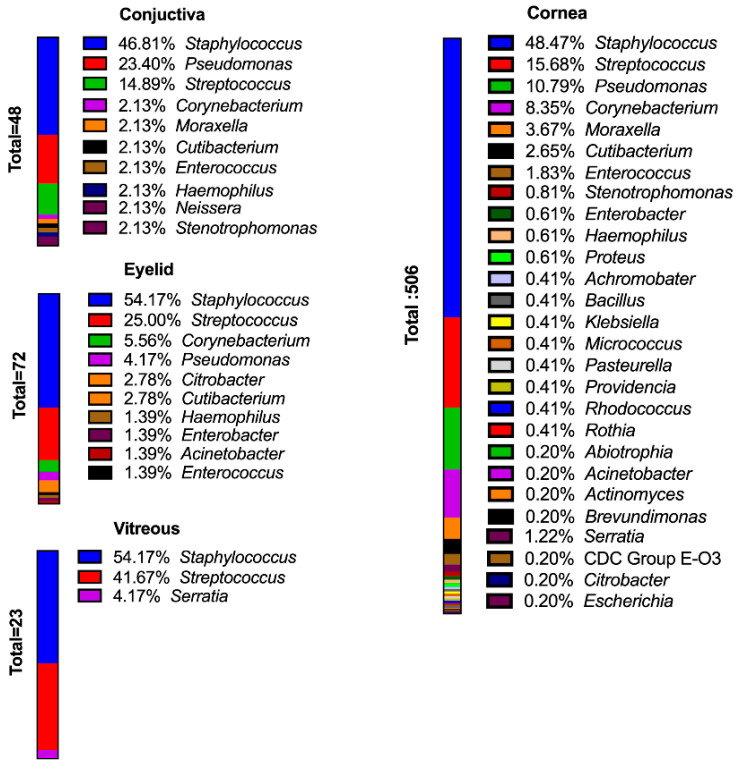
Distribution of bacterial genera and species at anatomic sites of the eye. Data are represented as percent of genera and species isolated from a particular ocular site.

Of the known anatomic sites sampled (Figure 1), the cornea was the source of 55.81% of the bacterial isolates. Eyelid (7.86%), conjunctiva (5.33%), and vitreous (2.55%) were also sampled. At all sampled sites, staphylococci were the predominant bacteria isolated (Figure 2 and Figure 3). Pseudomonads were the most common Gram-negative bacterial isolate, except in vitreous, where *Serratia* was the most common Gram-negative bacterial isolate. These data generally agree with those of Armstrong [1], Lakhundi et al. [11], and Nair et al. [12], where staphylococci and *Pseudomonas* were most commonly isolated from ocular infections.

## 2. Species Distribution of Isolates

**Figure 4:**
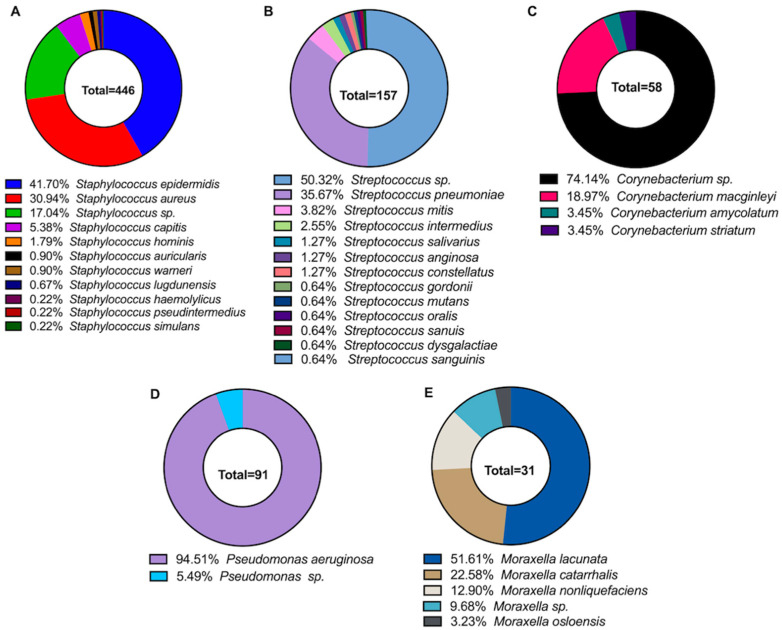
Distribution of specific species within a genus of ocular bacterial isolates. Data are represented as a percent of isolates of *Staphylococcus* (**A**), *Streptococcus* (**B**), *Corynebacterium* (**C**), *Pseudomonas* (**D**), and *Moraxella* (**E**) noted in this survey.

Among the Gram-positive bacteria, the most common staphylococcal species was *Staphylococcus epidermidis* (*S. epidermidis*) (41.70%)*. Staphylococcus aureus* (*S. aureus*) (30.94%) was the next most common (Figure 4A). *Streptococcus* sp. (unidentified species) (50.32%) and *Streptococcus pneumoniae* (*S. pneumoniae*) (35.67%) were the most common streptococcal species in the collection (Figure 4B). *Corynebacterium* were also frequently isolated but were generally not speciated and may have been part of the normal microbiota [13,14]. *Corynebacterium macginleyi* (18.97%) was the most commonly identified *Corynebacterium* species isolated from ocular tissues (Figure 4C). The pseudomonad isolated with the greatest frequency was *Pseudomonas aeruginosa* (*P. aeruginosa*) (94.51%), and the *Morexella* species isolated most frequently was *Moraxella lucunata* (*M. lucunata*) (51.61%) (Figure 4D,E respectively).

As discussed above, numerous species have been isolated from the surface of the eye. Expectations for isolating a particular group of organisms in an infection are useful, but to make rational care decisions, it is also necessary to know the pathogenic potential of these isolates. Using our survey of ocular isolates as a basis, we provide a review of the basic bacteriology and pathogenic potential of each genus of bacterial isolates, from the most common isolates to those only isolated once (*Hapax Legomenon*) during the time period of our survey. In this review, we also describe the range of ocular infections these genera are known to cause and their virulence factors, pathogenic potential, and treatment options.

## 3. Gram-Positive Ocular Pathogens

### 3.1. Bacillus

*Bacillus* is a Gram-positive, motile, spore-forming rod-shaped bacteria ubiquitously present in nature and is found most commonly in the soil [15]. *Bacillus anthracis* and *Bacillus cereus* are the two major *Bacillus* species that are medically significant. *B. anthracis*, the causative agent of anthrax, has historically gained the most attention. *B. cereus* is well known for its association with food poisoning, and *Bacillus* species other than *anthracis* are also associated with various systemic and acute infections [16]. Compared to other anatomical sites where *Bacillus* causes disease, the eye is extremely sensitive to this pathogen. The reason is two-fold: the eye is an immune-privileged site and inflammation is particularly dangerous to this organ, and *B. cereus* contains a particularly potent virulence arsenal. *Bacillus* has been reported to be associated with blinding forms of keratitis, post-traumatic endophthalmitis, and endogenous endophthalmitis. *Bacillus* keratitis is relatively rare and is commonly associated with ocular trauma and other ocular surface disturbances [17]. To our knowledge, there have been only two reports of *Bacillus* keratitis associated with contact lens use [18,19]. In ophthalmology, there are valid concerns about *Bacillus* spore contamination of eye makeup [20] and decorative contact lenses [21]. Although *B. cereus* is the most commonly encountered species in ocular infections, other *Bacillus* species have been recovered from keratitis; *B. megaterium* [17,22], *B. subtilis* [17,18], *B. coagulans, B. firmis, B. lincheniformis*, and *B. polymyxa* [17]. In an analysis of 39 *Bacillus* ocular isolates [23], 52.6% were *B. cereus*, 26.3% were *B. thuringiensis*, and other isolates included *B. subtilis*, *B. mycoides*, *B. pumilus*, *B. flexus*, and *Paenibacillus polymyxa* (formerly *Bacillus polymyxa*). Among all the organisms that cause endophthalmitis, members of the *Bacillus cereus sensu lato* group cause the most severe form of this disease that results in loss of vision in less than three days in most cases [24]. In addition to its motility, *B. cereus* possesses adhesive pili [25] and produces numerous toxins and enzymes which are under the control of the quorum-sensing regulator PlcR [25]. *Bacillus* toxins produced in mouse eyes during experimental endophthalmitis include hemolysin BL, nonhemolytic enterotoxin, cereolysin O, and enterotoxins A, C, and FM [26]. *Bacillus* rapidly replicates in the ocular environment during an active infection [25]. Therefore, proper treatment should be initiated as soon as possible [27]. *B. cereus* is inherently beta-lactam resistant but is sensitive to vancomycin. The empiric treatment of *Bacillus* endophthalmitis includes intravitreal injection of vancomycin and ceftazidime [28]. In experimental models of endophthalmitis, gatifloxacin is able to sterilize rabbit and mouse eyes infected with *Bacillus* [29,30]. Our survey included six isolates of *Bacillus* sp.: three from unlisted locations, one from contact lenses, and two from the cornea.

### 3.2. Corynebacterium

*Corynebacterium* is a genus composed of rod-shaped or coccobacilli bacteria with a club-shaped appearance, and its members are widely distributed among animals and plants [13]. Some species of *Corynebacterium* are rare opportunistic pathogens, primarily in immunocompromised individuals [13]. Species that have been isolated from cases of conjunctivitis or keratitis are *Corynebacterium accolens, C. amycolatum*, *C. mastitidis*, *C. propinquum*, *C. pseudodiphtheriticum*, *C. striatum*, and *C. xerosis* [14,31,32,33,34,35]. By far, the most commonly isolated *Corynebacterium* reported in ocular infections is *C. macginleyi* [14,35].

*Corynebacterium macginleyi* is a lipophilic facultative anaerobic rod that has been commonly isolated from healthy eyes [35]. Hoshi et al. [36] sampled the conjunctiva of patients prior to cataract surgery and reported that 84% of the *Corynebacterium* isolates were *C. macginleyi*. Although *C. macginleyi* has been isolated from various types of infections, such as endocarditis, surgical site infections, and bladder catheter infections [37,38,39], the majority of *C. macginleyi* case reports are of ocular infections [14]. *C. macginleyi* has been isolated from cases of blebitis, conjunctivitis, endophthalmitis, and keratitis [35,40,41,42,43]. *C. macginleyi* has few known virulence factors, but this may be due to the lack of functional studies conducted with this species. Sagerfors et al. [35] reported that 37% of *C. macginleyi* genes have unknown functions. In their study of 29 culture-proven cases of *C. macginleyi* keratitis, this group also reported that the course of the infections was “uneventful”, with no need for a corneal transplant. However, more serious cases required corneal cross-linking and amniotic membrane transplant [35]. The most common risk factors for *C. macginleyi* keratitis were contact lens wear (66%) and ocular surface disease (10%) [35].

The antibiotic resistance of *C. macginleyi* varies depending on the report: Sangerfors et al. [35] reported that all *C. macginleyi* isolates in their study were susceptible to fluoroquinolones, while Eguchi et al. [41] reported that 68.8% of their *C. macginleyi* isolates had “high levels of resistance” to all fluoroquinolones tested. Aoki et al. [14] observed that the antibiotic resistance of *C. macginleyi* varies by region and stated that *Corynebacterium* species remain susceptible to third-generation cephems, which they recommended as a pragmatic treatment for ocular infection caused by *Corynebacterium* species.

St. Leger and Caspi [44] isolated *Corynebacterium mastitidis* (*C. mastitidis*) from the conjunctiva of a group of C57BL/6 mice housed at the National Institutes of Health and demonstrated that *C. mastitidis* is able to form a stable ocular colonines in mouse eyes. This species has been used in mouse models to study how the ocular microbiome affects the ocular surface immune homeostasis [45]. To our knowledge, this organism has not been isolated from human ocular infection.

In our survey, there were 58 isolates of *Corynebacterium* which comprised 6.42% of all the isolates (Figure 2). The most common anatomical site of isolation was the cornea, of which 8.35% of all corneal isolates where *Corynebacterium* (Figure 3). A total of 74.14% of the *Corynebacterium* were only identified to the genus, 18.97% (11 isolates) of the isolates were *C. macginleyi*, two were *C. amycolatum*, and two were *C. striatum* (Figure 4C).

### 3.3. Cutibacterium acnes (Propionibacterium acnes)

*Cutibacterium acnes* (*C. acnes*), formerly called *Propionibacterium acnes*, is an aerotolerant anaerobic, rod-shaped bacterium that is part of the microbiota of the oral cavity, conjunctiva, and skin, and is most commonly known as the causative agent of acne vulgaris [46,47]. *C. acnes* has also been reported as part of the conjunctival microbiota [48]. With regards to eye infections, *C. acnes* has been isolated in cases of conjunctivitis, cellulitis, infectious keratitis [49,50], and in delayed-onset post-surgical endophthalmitis following cataract surgery [51,52]. *C. acnes* has a number of virulence factors, such as Christie-Atckins–Munch–Petersen factors (CAMP factors), porphyrins, hyaluronate lyase, adhesins, and the ability to form biofilms [53]. *C. acnes* is also naturally resistant to 5-nitromidazole agents, aminoglycosides, sulfonamides, mupirocin, and resistance to erythromycin and clindamycin is developing [53]. Isolates of *C. acnes* remain sensitive to vancomycin and β-lactams [49]. 

Because of their protracted time course, descriptions of *C. acnes* ocular infections commonly include words like “slow”, “chronic”, “indolent”, “smoldering”. In their review of *C. acnes* endocarditis, Gunthard et al. [54] reported that the average time needed to detect growth in anaerobic or aerobic cultures was 6 days, with a range of 2–15 days. *C. acnes* keratitis is characterized by small lesions with deep stromal infiltration in the peripheral cornea, and it has been suggested that *C. acnes* should be considered in cases of negative keratitis cultures after seven days of incubation [50]. In their retrospective review of six cases of *C. acnes* endophthalmitis following cataract extraction with intraocular lens implantation, Fowler et al. [52] reported that the average time from surgery to diagnosis was 7.4 ± 5.2 months. In the same study, 100% of patients who underwent intraocular lens (IOL) removal achieved complete resolution of their endophthalmitis, compared to 77% of those undergoing pars plana vitrectomy with partial capsulectomy plus intravitreal antibiotics, or 18% receiving antibiotics alone. In general, visual outcomes of *C. acnes* endophthalmitis are typically good, but IOL removal may be necessary. 

In our survey, there were 21 isolates of *C. acnes*, which comprised 3.21% of all isolates collected (Figure 2). A total of 47.6% of the isolates were from unlisted anatomical locations, 42.8% were collected from the cornea, and 1 isolate was collected from the conjunctiva.

### 3.4. Enterococcus

*Enterococcus* are facultative cocci known for their ability to grow and thrive under a variety of harsh environmental conditions, such as high salinity, low pH, and temperatures ranging from 10 to 45 °C [55]. The species *E. faecalis* and *E. faecium* rank among the leading causes health-care associated infections, including include urinary tract infections, bacteremia, surgical site infections, and endocarditis [55]. *Enterococcus* is a feared pathogen because of frequent antibiotic resistance to aminoglycosides, β-lactams, and vancomycin [56]. *E. faecalis* accounts for 4 to 8% of cases of post-operative endophthalmitis, including filtering bleb infections following glaucoma surgery [57,58,59,60,61]. The visual outcome of endophthalmitis due to *E. faecalis* is uniformly poor [57]. *E. faecalis* possesses several virulence factors, such as aggregation substance, enterococcal surface protein, hemolysins, extracellular superoxide, and gelatinase [62]. Cytolysin is one of the most important enterococcal virulence factors [63]. Cytolysin is a pore-forming exotoxin capable of lysing bacterial and eukaryotic cells [63], and this toxin may be responsible for poor visual outcomes in *E. faecalis* endophthalmitis [55,64]. *E. faecalis* endophthalmitis, as well as other types of *E. faecalis* infections, are increasingly caused by strains resistant to multiple antibiotics, including the last resort drug, vancomycin [55,60,65]. As such, *E. faecium* is a member of the “ESKAPE” group of bacterial pathogens [66]. Because of the increasing threat of multi-drug resistant infections, determining the mechanisms by which *E. faecalis* causes intraocular infection is vital. 

Our survey contained 15 *Enterococcus* isolates: 11 *E. faecalis*, three nonspeciated enterococci, and one *E. cloacae*. Of the 11 *E. faecalis* isolates, six were isolated from the cornea, one each from the conjunctiva and eye, and three were from unlisted locations. The single *E. cloacae* isolate was from the cornea. *Enterococcus* comprised 1.66% of all the species isolated in this study (Figure 2).

### 3.5. Micrococcus

*Micrococcus* is a Gram-positive coccus in the Micrococcaceae family and is widely found in water, soil, dust, on the skin, and in the conjunctival microbiota of humans [67]. *Micrococcus luteus* (*M. luteus*) is the species most commonly isolated from human skin and infections, with *Micrococcus lylae* being more rarely isolated from human skin [68]. Although *M. luteus* is not considered to be pathogenic, there have been reports of various types of infections associated with this organism in the immunocompromised, such as HIV patients [69]. *M. luteus* has been associated with infections, including septic shock [70], meningitis [71], and catheter infections [72]. Because it is a commensal, the virulence of *M. luteus* has not been widely studied, but this species is capable of forming biofilms on implanted medical devices [73]. Although *M. luteus* is usually penicillin–sensitive, biofilm formation can make infections with this commensal more resistant to antibiotic treatment. In those cases, vancomycin and rifampin are recommended for their ability to penetrate biofilms [73]. The association of *Micrococcus* with ocular infection is rare. *M. luteus* was reported as the cause of keratitis in the left eye of a patient who underwent simultaneous LASIK for myopia [67]. The isolated *M. luteus* was resistant to ciprofloxacin and oxacillin and was treated successfully with fortified cefazolin 5% [67]. 

*Micrococcus* was an uncommon isolate in this survey, with only three isolates collected, none of which were identified to the species level. Two isolates were collected from the cornea, and one was collected from an unlisted location. *Micrococcus* comprised only 0.33% of all the genera collected in our survey (Figure 2).

### 3.6. Staphylococci

#### 3.6.1. *Staphylococcus aureus*

*Staphylococcus aureus* (*S. aureus*) is a nonmotile coccus that occurs in irregular grape-like clusters. *S. aureus* is the most significant pathogen of the staphylococci group and is a common cause of food poisoning [74], abscess formation, pyogenic infections, and fatal septicemia [75]. *S. aureus* are characterized by a β-hemolytic phenotype, a positive coagulase reaction, mannitol fermentation, and the golden pigmentation from which their species name is derived [76]. A positive coagulase reaction separates *S. aureus* from the other staphylococci species isolated from humans (the coagulase-negative staphylococci, CoNS) [77]. *S. aureus* are part of the human microbiota and have been isolated from the nasal mucosa of a quarter to one-third of healthy individuals [78]. *S. aureus* has been reported to comprise 1.8% to 25% of species cultured from healthy eyes (Table 1). In our survey, there were 138 *S. aureus* isolates, comprising 15.39% of all the isolates collected and 30.94% of all staphylococcal isolates (Figure 4A). Additionally, 55.8% of the *S. aureus* isolates were isolated from the cornea, 6.5% were conjunctival isolates, and 19.62% were from unlisted locations. 

*S. aureus* is a leading cause of a host of eye infections, such as blepharitis, cellulitis, conjunctivitis, keratitis, dacryocystitis, and endophthalmitis [5,6,9,27,79,80,81,82,83,84,85]. Because of its potential for multidrug antibiotic resistance and the impressive array of toxins and enzymes in its arsenal, *S. aureus* is considered a formidable and often dangerous pathogen [86,87]. 

**Table 1:** Isolation of normal ocular flora from different retrospective studies across the world.

	*Location*	*Study Population*	*Gram-Positive*	*Bacillus*	*CoNS*	*S. epidermidis*	*S. aureus*	*Gram-Negative*	*Pseudomonas*	*P. aeruginosa*
**Arantes [88]**	Brazil	Pre-cataract	88.90%		54%		8%	11.10%		
**Capriotti [89]**	Sierra Leone	Healthy Individuals	78.1%	5.5%	36.1%		25.1%	21.9%		7.8%
**Dave [90]**	USA, Nashville	Intravitreal Injections				45.70%	6.50%	8.70%		
**Dorrepaal [91]**	Toronto	Intravitreal Injections			64%		1.80%	0.90%		
**Hsu [92]**	USA, St. Louis	Pre-cataract	90.5%		74.8%	57.2%	5.0%	9.5%	1.8%	
**Lin [93]**	Taiwan	Pre-cataract	91.70%		45.20%	16.70%	2.40%	8.30%	4.80%	
**Mamah [94]**	Nigeria	Pre-cataract	73.70%		34.20%	34.20%	13.20%		2.60%	
**Martins [95]**	Sao Paulo	Healthy Individuals			61.70%		11.70%		3.40%	
**Mshangila [96]**	Uganda	Pre-cataract			65.90%	76.90%	21.00%	10.10%		
**Papa [97]**	Italy	Pre-cataract	95%		67.90%	58.00%	15.30%	4.60%		
**Rubio [98]**	Madrid	Pre-cataract			56.80%		6.40%	7.30%		
**Suto [99]**	Japan	Pre-cataract	67%			57.20%	3.90%	6.30%	0.70%	

We conducted virulence gene analysis by PCR for all of the *S. aureus* ocular isolates in this survey. The PCR primers are included in Table 2, and isolated DNA preparations of these isolates were subjected to PCR as described in [10]. Ninety-eight percent of all *S. aureus* isolates were positive for *hla* (Table 3), the gene coding for α-toxin. Other studies have reported that 95–100% of clinical isolates were positive for *hla* [10,86]. α-toxin is also known as α-hemolysin because of its ability to produce β-hemolysis on blood agar [100]. α-toxin is a beta-barrel pore-forming toxin that binds to the receptor of a disintegrin and metalloproteinase 10 (ADAM10) [101]. High concentrations of α-toxin result in cell death, but sublytic concentrations of the toxin binding to ADAM10 activate ADAM10 metalloprotease activity, causing cleavage of E-cadherin adherens junctions, resulting in disruption of cellular focal adhesions and tissue breakdown [101]. It is not known if the ADAM10-mediated tissue damage mechanism occurs when *S. aureus* infects the cornea. However, in a rabbit model of keratitis, isogenic mutants of *S. aureus* lacking α-toxin injected into corneas caused no epithelial erosions as did the α-toxin-producing parental strain [102]. Similar studies conducted in a mouse model of keratitis confirmed that an α-toxin mutant was less virulent for the cornea [103]. In that study, corneal pathology caused by *S. aureus* was more severe in aged mice (36–48 weeks old) compared to young mice (6–7 weeks old) [103]. Putra et al. [104] used *S. aureus* strain JE2 in a corneal debridement model of keratitis in mice in which corneal healing was more rapid following infection with the α-toxin-deficient mutant strain compared to that of the parental strain. In a rabbit model of *S. aureus* endophthalmitis, infection with the isogenic α-toxin-deficient mutant caused less retinal damage than the parental strain [27]. These findings were later confirmed in a mouse model of *S. aureus* endophthalmitis [105]. These results indicate that α-toxin plays a major role in *S. aureus* ocular virulence. This toxin can contribute to pathogenesis either by direct killing of cells and/or by stimulation of the immune response. Blocking the activity of α-toxin would therefore be a rational therapeutic target for improving the visual outcome of keratitis and endophthalmitis. Theoretically, this could be achieved by passive or active anti-toxin immunization or the use of nanoparticles to neutralize the pore-forming toxins [29,106].

The percentage of *S. aureus* isolates in the current study that were positive for *hlb*, the gene encoding β-toxin, was 77.5% (Table 3), with 75% of *mecA* positive isolates also possessing the β-toxin gene. Reports of β-toxin frequency in clinical isolates ranged from 39% [10] to 57% [108]. β-toxin is not a toxin per se but is a neutral sphingomyelinase that hydrolyzes the plasma membrane lipid sphingomyelin, producing α-hemolysis on blood agar plates [109]. Most strains of *S. aureus* do not produce β-toxin due to the insertion of phage φSa3 into the *hlb* gene [110]. However, during in vivo growth, the phage φSa3 can excise and restore β-toxin production [111]. The role of β-toxin in *S. aureus* keratitis is unclear. In a rabbit model of *S. aureus* keratitis, infection with isogenic β-toxin-deficient mutants resulted in less scleral edema than that observed during infection with the wild-type strain. However, epithelial erosions, intrastromal ulcers, and slit lamp scores were similar in infections caused by *hlb*-deficient mutants [112]. In the rabbit model of experimental endophthalmitis, injection of an isogenic β-toxin-deficient mutant resulted in significantly less retinal damage compared to the wild-type parental strain [27]. These studies suggest that β-toxin may play a role in the virulence of *S. aureus*, but damaging activities might be more apparent in the posterior segment. 

Panton–Valentine leucocidin (PVL) is a prophage-encoded toxin that binds to the C5a receptor, targeting neutrophils, monocytes, macrophages, natural killer cells, dendritic cells, and T-lymphocytes [113]. The percentage of *S. aureus* positive for PVL-associated genes in non-ocular clinical samples was reported to be 1.6% to 10% [114,115]. The percentage of all *S. aureus* positive for PVL-associated genes in staphylococcal isolates in our survey was 23.3% (Table 3). Of the *mecA*-positive isolates in our study, 37.5% were positive for PVL-associated genes. Bispo et al. [116] examined 68 isolates of MRSA from keratitis and orbital abscess/cellulitis cases and reported that the isolates grouped into two clonal complexes, CC5 and CC8. Isolates in the CC8 linage primarily caused orbital abscess/cellulitis and were predominately composed of USA300 strains in which *pvl* is more common, while CC5 primarily caused keratitis cases and was populated by USA100 and USA800 strains in which *pvl* was less common [116]. CC8 isolates were 93.7% positive for *pvl*, and none of the CC5 isolates were positive for *pvl* [116]. The role of PVL in ocular infections is unclear. Foster et al. [117] studied isolates from 85 cases of pediatric periorbital or orbital cellulitis and reported that 85% were *pvl* positive and 78% of these 85 cases were USA300 strains. This group further reported that there was no difference in clinical features or visual outcomes comparing infections with *pvl* positive or negative isolates or USA300 to non-USA300 isolates [117]. Sueke et al. [118] examined 95 keratitis isolates and reported that 9.5% were *pvl*-positive, and the *pvl*-positive cases suffered larger corneal ulcers and required more surgical intervention than *pvl*-negative cases.

*S. aureus* is often multidrug-resistant and is a member of the ESKAPE group of bacterial pathogens. In the antibiotic resistance monitoring in ocular microorganisms (ARMOR) study in the United States, ocular isolates of *S. aureus* were resistant to azithromycin (60.6%), ciprofloxacin (35.8%), and methicillin (36.6%); fewer were also resistant to chloramphenicol (6.1%), trimethoprim (4.4%), tetracycline (4.3%); and all isolates were sensitive to vancomycin [119]. Multidrug resistance was high among *S. aureus*, with 32.0% of isolates being resistant to three or more drug classes [118]. Bispo et al. [116] reported that 26.7% of their ocular isolates were MRSA. In our survey, of the 129 *S. aureus* ocular isolates tested, 37.2% were *mecA* positive (Table 3).

#### 3.6.2. Coagulase-Negative Staphylococci

The coagulase-negative staphylococci (CoNS) group includes more than 50 species of staphylococci, whose species are distinguished from *S. aureus* by their inability to coagulate plasma [120]. Most members of the CoNS group cause chronic rather than life-threatening acute infections, but because of the high frequency of these infections, the difficulty diagnosing the etiology of an infection with a commensal bacterium, and the high rate of antibiotic resistance in this group, CoNS infections can be a burden on health care systems and have a profound impact on patient health [120]. Several CoNS species have emerged as pathogens of health-care facilities: *Staphylococcus capitis*, *S. epidermidis*, *S. haemolyticus*, *S. lugdunensis*, and *S. saprophyticus* [121]. *Staphylococcus epidermidis* (*S. epidermidis*) is the most commonly isolated CoNS in clinical samples and is the most widely studied of all CoNS [120,121]. Although *S. epidermidis* lacks the classical *S. aureus* virulence factor α-toxin, *S. epidermidis* isolates produce many potential virulence factors such as metalloproteases, β-hemolysin, δ-hemolysin, phenol-soluble modulins, proteases, numerous adhesion factors, and can form biofilms [121]. Antibiotic resistance is widespread in CoNS, and the group may serve as a reservoir of antibiotic-resistance genes for *S. aureus* [122]. The propensity of CoNS to form biofilms and their widespread antibiotic resistance make these infections difficult to treat [118,123,124]. 

CoNS are the most frequently isolated cause of many ocular infections, such as post-injection, post-operative, and post-traumatic endophthalmitis [51] and keratitis (Table 4). Patients recovering from endophthalmitis caused by CoNS are more likely to recover baseline visual acuity than those recovering from endophthalmitis caused by *S. aureus* or *Streptococcus* sp. [125]. Among surveys of bacteria from healthy eyes, CoNS comprise 34% to 74.8% of all bacterial isolates (Table 1). Among keratitis isolates, CoNS comprise 5% to 48% of bacterial species isolated. In our survey, 34.1% of all isolates were CoNS, with 44.8% of those being *S. epidermidis*. Eight different species of CoNS were isolated in this survey (Figure 4A), with 24.68% not identified at the species level (Figure 4A). 

Since CoNS are part of the ocular microbiota, their identification as pathogens should be considered in view of the individual patient history [120]. Isolation of CoNS from the eyes of immunocompromised patients, such as those with poorly control diabetes, cancer patients, and chronic ocular corticosteroid use, should be considered in the keratitis diagnosis [2]. The ARMOR study noted above reported that ocular CoNS were resistant to azithromycin (61%), methicillin (48.6%), and ciprofloxacin (34.9%), and had less frequent resistance to tobramycin (17.0%), tetracycline (13.9%) and chloramphenicol (1.2%). All isolates were sensitive to vancomycin [119]. Multidrug resistance was also high among CoNS, with 40.7% of isolates being resistance to three or more drug classes [119]. Most of the CoNS in the ARMOR study were *S. epidermidis*.

**Figure 5:**
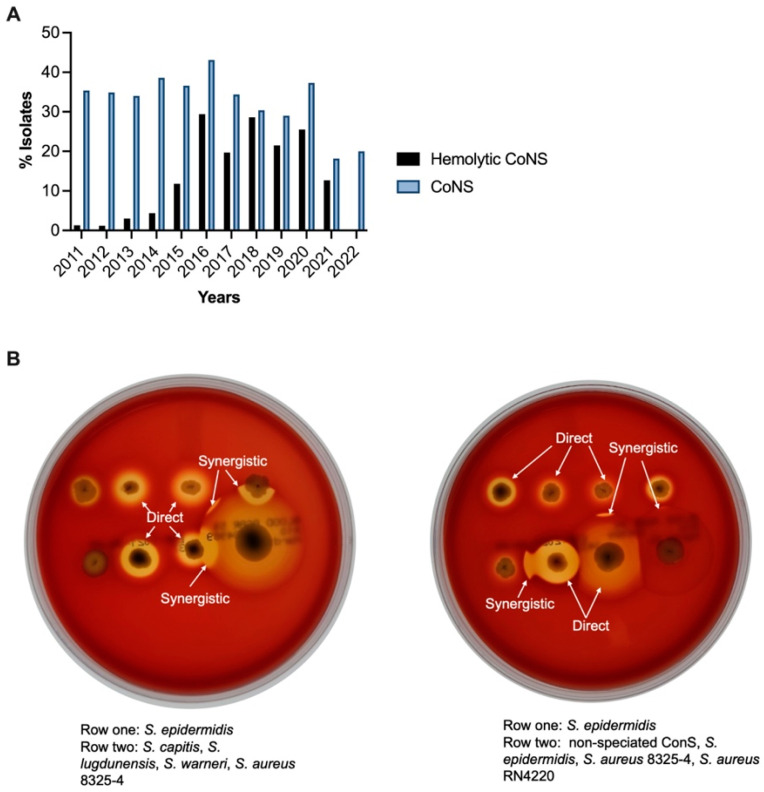
(**A**) Distribution of hemolytic coagulase-negative staphylococci (CoNS) compared with CoNS isolates over a ten-year span. Data are demonstrated as percent CoNS in the total isolate pool and percent hemolytic CoNS in the total isolate pool. (**B**) Representative examples of the variety of hemolysis phenotypes of CoNS isolates after overnight incubation at 37 °F on 5% sheep blood agar. Direct hemolysis surrounding a colony is likely derived from synthesis of a hemolytic toxin or enzyme from growing CoNS, while synergistic hemolysis between colonies may be derived from the interaction of one or more of these hemolysins. Hemolysis of *S. aureus* lab strains 8325-4 and RN4220 are also shown for comparison.

Although CoNS are classically described as being nonhemolytic [138,139], there are reports of a β-hemolytic phenotype among members of this group [140,141,142,143,144]. The α-toxin gene *hla* has been detected by PCR in *S. epidermidis* [141,143,144,145]. Okee et al. [141] suggested that the cause of the hemolytic phenotype in these strains may be caused by a combination of factors. In their study of community and ICU isolates of *S. epidermidis*, this group reported that 70% of isolates from their ICU were β-hemolytic on 5% sheep blood agar, but only 20% were *hla* positive by PCR [141]. Interestingly, this phenotype was only detected in hospital-acquired isolates; no isolates from the community were β-hemolytic or *hla* positive. In the first year of our survey (2011), 35% of all isolates were CoNS, and only 3.57% of CoNS were β-hemolytic. In the last full year of our survey (2021), 18.2% of all isolates were CoNS, but 70% of these CoNS were β-hemolytic (Figure 5A). Over the 10-year time span, 40.7% of all CoNS in our survey had a β-hemolytic phenotype. Examples of hemolytic and non-hemolytic CoNS are shown in Figure 5B, illustrating the variation in hemolytic zones by these isolates compared with the hemolysis produced by β-hemolytic and non-β-hemolytic *S. aureus* laboratory strains (strains 8325-4 and RN4220, respectively). The origin of this evolving and potential pathogenic phenotype is under investigation.

#### 3.6.3. *Staphylococcus pseudintermedius*

There was a single isolate of *Staphylococcus pseudintermedius* (*S. pseudintermedius*) noted in our survey (Figure 4), isolated from the cornea. *S. pseudintermedius* is a coagulase-positive *Staphylococcus* that has been isolated from 20.9% of healthy dogs [146] and is the most common staphylococcal species isolated from dogs [147]. *S. pseudintermedius* has been reported as an emerging zoonosis of canine origin and has been reported as the cause of skin and soft tissue infections in humans [148]. *S. pseudintermedius* has a high rate of methicillin resistance and multidrug resistance [148,149,150]. There are five main clonal lineages of methicillin-resistant *S. pseudintermedius*, each of which has distinct antimicrobial resistance profiles, geographic distributions, and *SCCmec* content [151]. We included *S. pseudintermedius* in this review as this species is a frequent isolate from canine ulcerative keratitis [149] and purulent soft tissue infections in canines and other domesticated animals [148,150]. *S. pseudintermedius* virulence mechanisms are not well studied but seem to be primarily due to the production of phenol-soluble modulins [152] and also include biofilm formation, lipase production, and toxins *hlgA* and *hlgB* [146]. As noted above, there is a high rate of multidrug resistance among *S. pseudintermedius*, but Ruiz-Ripa et al. [147] reported that all isolates in their study were susceptible to vancomycin and linezolid.

### 3.7. Streptococci

On lists of the prevalence of ocular bacterial isolates, streptococci are commonly ranked third, after CoNS and *S. aureus*, if they are listed at all. *Streptococcus* are Gram-positive bacteria that grow in chains or pairs and are commensals in the upper respiratory tracts and gastrointestinal tracts of most mammals and birds [153]. *Streptococcus* can become opportunistic pathogens under suitable conditions, such as in infections in elderly or immunocompromised patients [153]. In studies of the culturable bacterial flora of healthy eyes, streptococci were reported to range from 0% in São Paulo [95] to 13.2% in Nigeria [94]. Haung et al. [154] and Shin et al. [155] detected *Streptococcus* by 16S rDNA from conjunctival samples of healthy individuals, indicating that *Streptococcus* forms part of the core microbiome of the conjunctiva. In our survey, 17.39% of all the isolated bacteria were streptococci (Figure 2). Eleven species of streptococci were identified, with *Streptococcus pneumoniae* (*S. pneumoniae*) being the most common, comprising 35.67% of the streptococci isolates (Figure 4B). Moreover, 50.32% of the streptococcal isolates were not identified at the species level (Figure 4B).

*S. pneumoniae* are the most commonly isolated streptococci from ocular infections such as conjunctivitis, endogenous and exogenous endophthalmitis, and keratitis [156,157]. *S. pneumoniae* is also the most common cause of bacterial keratitis in low-income countries [158]. Among keratitis isolates, *S. pneumoniae* has been reported to have been isolated in 2% to 46.80% of cases (Table 4). In cases of endophthalmitis, even with prompt treatment, there is a high risk of profound vision loss and enucleation or evisceration in eyes infected with *S. pneumoniae* [156,157]. Chen et al. [157] reported that of 38 cases of *S. pneumoniae* endophthalmitis, 84% resulted in light perception to no light perception, and 26% underwent evisceration or enucleation. 

*S. pneumoniae* possesses a number of virulence factors, such as a polysaccharide capsule, neuraminidase, pneumolysin, and zinc metalloproteinases [157]. The polysaccharide capsule enables *S. pneumoniae* to evade phagocytosis by inhibiting complement-mediated opsonization and is found in the majority of invasive isolates [159]. In animal models, encapsulated and nonencapsulated strains of *S. pneumococcus* were capable of causing severe keratitis [160], and the capsule was shown to be necessary for full virulence in the rabbit model of endophthalmitis [156]. The pore-forming toxin pneumolysin is highly conserved among pneumococcal isolates, and in addition to being cytotoxic to corneal epithelial cells, pneumolysin is highly immunogenic and causes an intense inflammatory response [161] and may be responsible for the rapid liquefactive necrosis that many pneumococcal ulcers undergo despite prompt treatment [3]. Endophthalmitis and keratitis caused by the *Streptococcus* genus as a whole are characterized by poor visual outcomes [162]. Gower et al. [163] reported that 70% *Streptococcus* sp. endophthalmitis cases following cataract surgery were count fingers or worse. 

In their study of 271 clinical conjunctivitis isolates, Valentino et al. [164] reported that 90% were nonencapsulated and formed a distinct clade characterized by divergent forms of virulence factors and adhesins not found in encapsulated strains. Andre et al. [165] studied 45 clinical keratitis isolates and reported that 95.2% were encapsulated, but the capsular types in these strains were not covered by the pneumococcal vaccine PCV-13. These isolates were sensitive to fluoroquinolones and vancomycin but showed varying degrees of resistance to macrolides, such as erythromycin and azithromycin. In the ARMOR study noted above, ocular *S. pneumoniae* isolates reported in vitro resistance to azithromycin (35.9%) and penicillin (33.3%), but resistance to fluoroquinolones was less than 1% [119].

## 4. Gram-Negative Ocular Pathogens

### 4.1. Achromobacter

*Achromobacter* is a multidrug-resistant rod-shaped bacteria found in soil and water that can cause a wide variety of opportunistic infections in immunocompromised patients, such as bacteremia, abscesses, meningitis, urinary tract infections, and pneumonia [166]. Nineteen species of *Achromobacter* have been described, with *Achromobacter xylosoxidans* (*A. xylosoxidans*) being the most commonly isolated species from clinical cases [167]. *Achromobacter* spp. are predominantly isolated from patients with cystic fibrosis [168,169]. Among patients not suffering from cystic fibrosis, *Achromobacter* pneumonia or bacteremia are the most common types of infection [169]. Species other than *A. xylosoxidans* demonstrate a geographical diversity, with *A. ruhlandii* being the second most commonly isolated species in North America. In Europe, *A. dolens* and *A. insuavis* are more common, but it is not known if these species are of clinical significance [170]. Because *Achromobacter* is infrequently isolated from human infections, its virulence factors, clinical features, and optimal treatments for *Acromobacter* infections are not well described [170]. *Achromocbater* produces biofilms, is motile, and is frequently multidrug-resistant [171], with intrinsic resistance to most cephalosporins, aztreonam, and aminoglycosides due to multidrug efflux pumps and chromosomal β-lactamases [170]. 

*A. xylosoxidans* is a rare cause of chronic conjunctivitis, keratitis, and post-surgical endophthalmitis [172,173,174]. In their retrospective review of 10 ocular infections caused by *A. xylosoxidans*, Reddy et al. [173] reported eight cases of keratitis, six of which developed following penetrating keratoplasty and two cases of endophthalmitis. The keratitis infections were characterized by a slowly progressive disease with localized infiltration [173]. This group also reported that 90% of their isolates were sensitive to ceftazidime, and 70% were sensitive to amikacin [173]. In our survey, two *Achromobacter* isolates were noted, one *A. xylosoxidans* from an unlisted location and one *Achromobacter* sp. isolated from the cornea. 

### 4.2. Acinetobacter

*Acinetobacter* are coccobacilli commonly found in soil and water samples and are frequently isolated from the skin of hospital staff and patients [175]. Although *Acinetobacter* are considered to be low-virulence opportunistic pathogens, this genus is capable of causing severe infections in immunocompromised patients following invasive procedures [175]. Although the danger of *Acinetobacter* lies in its high level of multidrug resistance, it is becoming appreciated that this species’ ability to adapt and survive lends to its persistence in hostile environments [176]. The presence of polysaccharide capsules in some *Acinetobacter* species, its ability to repair its genome during rehydration, and its high tolerance to oxidative stress all contribute to its desiccation resistance [176]. *Acinetobacter* also forms biofilms, is motile, and uses its capsule to circumvent host immunity. *Acinetobacter* are becoming increasingly resistant to several antibiotics and, as such, is a member of the ESKAPE group of bacterial pathogens [66]. Talreja et al. [177] reported that all 12 of the ocular *Acinetobacter baumannii* isolates tested in their study were multidrug resistant. *Acinetobacter anitratus* [178] and *A. lwoffi* [179] have been reported as rare causes of post-traumatic endophthalmitis, and *Acinetobacter baumannii* [177] and *A. junii* [180] have been reported as the cause of corneal ulcers. In our survey, three *Acinetobacter lwoffi* isolates were noted and were isolated from the cornea, aqueous, and an unknown region of the eye.

### 4.3. Citrobacter

*Citrobacter* sp. are rod-shaped common environmental bacteria comprised of 11 recognized species that have been found in the normal gut microbiota of humans [181,182]. Citrobacter are an increasing problem in human infections, such as urinary tract infections and bacteremia, because of evolving multidrug resistance [183]. Common virulence and adaptation traits, such as polysaccharide capsules, iron acquisition genes, and motility operons, have been reported in this genus, as well as the presence of a high-pathogenicity island that is essential for virulence in mice [181]. *Citrobacter freudii* (*C. freudii*) and *Citrobacter koseri* (*C. koseri*) have been reported in cases of exogenous and endogenous endophthalmitis [183,184] and keratitis [185,186,187]. Overall, reports of *Citrobacter* in eye infections have been relatively rare but almost always resulted in severe infections and vision loss [184]. In our survey, we report four *Citrobacter* isolates: two *C. koseri* from an unknown area of the eye and one from the cornea, and a *C. freudii* isolate from an unknown area of the eye.

### 4.4. Enterobacter

*Enterobacter* are rod-shaped, facultative bacteria and are members of the family *Enterobacteriaceae* [188]. The *Enterobacter* genus consists of 22 species, some of which are members of the normal gastrointestinal microbiota, but can cause healthcare-associated infections such as urinary tract, respiratory, and soft tissue infections, as well as osteomyelitis and endocarditis, especially in immunocompromised individuals [188]. The virulence factors of *Enterobacter* are poorly understood but include motility, chemotaxis, and capsules [189]. Because of their evolving multidrug resistance, *Enterobacter* is a member of the ESKAPE group of bacterial pathogens [66]. While intraocular infections with *Enterobacter* are rare, cases of postoperative and posttraumatic endophthalmitis have been reported [190,191,192]. *E. cloacae* has been associated with acute postoperative, delayed filtering bleb-related, and posttraumatic endophthalmitis [190,191,192]. In their retrospective study of 44 culture-positive cases of *Enterobacter* endophthalmitis, Dave et al. [193] reported that 77.27% were from posttraumatic cases, 15.9% were from postoperative cases, and 6.8% were from endogenous endophthalmitis cases. Isolates from cases of *Enterobacter* endophthalmitis were reported to be susceptible to ciprofloxacin, amikacin, and ceftazidime [193,194]. *Enterobacter*-associated endophthalmitis uniformly presents as a rapid and severe infection and results in poor visual outcomes despite early and appropriate management [193]. In our survey, seven *Enterobacter* isolates are reported, five *E. cloacae* (two from the cornea, one from the eye, and two from unlisted locations), one *E. cancerogenus* (from the cornea), and one *E. aerogenes* (from the lacrimal gland).

### 4.5. Escherichia coli

*Escherichia coli* (*E. coli*) is a Gram-negative, facultatively anaerobic, motile, non-spore-forming rod-shaped bacteria [195]. *E. coli* are mainly commensal members of the large intestine, but certain strains of this species are pathogenic and are classified into pathotypes based on various criteria, such as the target organ, host species, or the presence of specific virulence genes [196]. Pathogenic *E. coli* can cause serious infections such as urinary tract infections, intra-abdominal, skin, and soft tissue infections, pulmonary infections, newborn meningitis, bacteremia, and hemolytic and uremic syndrome [196]. *E. coli* possess numerous virulence factors encoded in pathogenicity islands, plasmids, and other mobile genetic elements [197]. These virulence factors include adhesins, toxins such as α-hemolysin and cytotoxic necrotizing factor 1, iron acquisition factors, polysaccharide capsules, and liposaccharide [197]. Antibiotic resistance in this species is widespread and increasing [196]. *E. coli* have been isolated from ocular infections such as conjunctivitis [5], dacryocystitis [198], keratitis [199,200], and endophthalmitis [200,201]. Jackson et al. [201] reported that seven percent of endogenous endophthalmitis cases were caused by *E. coli*, which is a rare but not uncommon complication of septicemia. In our survey, two *E. coli* isolates were noted, one from the cornea and one from an unlisted location in the eye.

### 4.6. Haemophilus

*Haemophilus* are Gram-negative coccobacilli that inhabit the upper respiratory tract and are rarely associated with ocular infections [202,203]. Pathogenic strains that cause disease generally enter the upper respiratory tract through droplet inhalation or direct contact [204]. The most common pathogenic *Haemophilus* are *Haemophilus influenzae* (*H. influenzae*), which are characterized by capsular type [205]. *H. influenzae* virulence factors include polysaccharide capsule, biofilm formation, IgA proteases, and macrophage survival factor [205]. *Haemophilus* are responsible for a range of mild and serious infections, including sinusitis, conjunctivitis, pneumonia, bacteremia, otitis media, meningitis, cellulitis, and epiglottitis [203]. Conjunctivitis is a common ocular bacterial infection occasionally caused by *H. influenzae* [206]. Conjunctivitis-otitis syndrome is a manifestation of acute conjunctivitis in infants that can be caused by *H. influenzae* [207]. Topical antibiotic therapy may reduce the duration of this disease, but it is typically self-limited within a few weeks. In the ARMOR study noted above, *H. influenzae* were “…susceptible to all antibiotics tested” [119]. In our survey, nine isolates of *Hemophilus* were collected, comprising 1% of all isolates (Figure 2). Eight of the isolates were *H. influenzae*, and one was not identified at the species level. Four of the isolates were from unlisted locations, three were from the cornea, and one each was from the conjunctiva and eyelid.

### 4.7. Klebsiella

*Klebsiella* species are non-motile, encapsulated rods and are predominantly opportunistic pathogens [208]. *Klebsiella* infections typically occur in hospital settings among individuals who are immunocompromised and have a severe underlying condition [209,210]. *K. pneumoniae* and *K. oxytoca* are responsible for the majority of healthcare-associated infections that include pneumonia, septicemia, soft tissue abscesses, meningitis, and endophthalmitis [208]. *K. pneumoniae* has become increasingly resistant to multiple antibiotics [211,212,213,214] and is a member of the ESKAPE group of bacterial pathogens [66]. The virulence of *K. pneumoniae* has been ascribed to the production of cell-wall-associated factors and capsules [215,216]. The hypermucoviscosity phenotype is commonly associated with strains that cause liver abscesses and those that possess enhanced intraocular virulence [217,218]. This hypermucoviscosity phenotype produces a mucopolysaccharide web which, in a mouse model of endophthalmitis, produced rapid retinal function decline and inhibited phagocytosis, as compared to the isotype mutant lacking this phenotype [216,217]. *K. pneumoniae* currently ranks among the leading causes of endogenous bacterial endophthalmitis and is responsible for 80–90% of cases in Asian countries [218].

In their review of 14 cases of endogenous *K. pneumoniae* endophthalmitis spanning 12 years, Mak et al. [219] reported that hepatobiliary sepsis was the source of ocular infection in 64% of patients, 14% of patients died, 38% experienced total loss of vision, and 19% required evisceration of the globe. The outcome of endogenous endophthalmitis is often severe, ranging from count fingers visual retention to evisceration or enucleation [218]. In a streptozocin-induced diabetic mouse model of endogenous *K. pneumoniae* endophthalmitis, intraocular infection incidence correlated with a compromised blood–retinal barrier and increases in vascular permeability [220]. Endogenous endophthalmitis was observed in mice 3 and 5 months following streptozocin injection but not in mice 1 month post-injection or in control, nondiabetic mice. These results suggested that *K. pneumoniae* requires a compromised blood–retinal barrier in order to gain access to the eye from the bloodstream and pointed towards an underlying mechanism for the increased prevalence of cases of *K. pneumoniae* endogenous endophthalmitis observed among diabetic patients [220]. In contrast, in a streptozocin-induced diabetic mouse model of endogenous *S. aureus* endophthalmitis, intraocular infections were observed in both nondiabetic mice, as well as in mice 1, 3, and 5 months post-streptozocin injection [221]. This indicated that *S. aureus* does not require a compromised blood–retinal barrier and can cause endogenous endophthalmitis in the absence of diabetes.

*K. oxytoca* has been reported as a rare cause of keratitis [222], and *K. pneumoniae* has been reported as a rare cause of interface keratitis following lamellar keratoplasty [223]. Although drug-resistant *K. pneumoniae* have been reported in Asia, 86% of endogenous bacterial endophthalmitis cases responded well to intravitreal ceftazidime [219]. In our survey, there were four isolates of *K. oxytoca* and one *K. pneumoniae*. Two of the isolates were from the cornea, and three were from unlisted locations.

### 4.8. Moraxella

*Moraxella* sp. are coccobacilli that were first reported from ocular infections in 1896 and 1897 from patients with angular blepharitis [224,225]. *Moraxella* are considered part of the microbiota of the upper respiratory tract and urogenital tract [226,227,228]. *Moraxella* sp. are known causes of keratitis [226,227,228,229], conjunctivitis [226,227,228,229,230], and endophthalmitis [226]. In their retrospective review of 101 culture-proven cases of *Moraxella* keratitis, Hoarau et al. [231] reported that the most common species were *M. lucunata* (50%) and *M. nonliquefasciens* (38%). This group also reported that the clinical features, such as ulcer size and healing, treatment duration, and infiltrate size, did not vary with the species causing the infection, and the preferred treatment was fluoroquinolone and rifamycin [231]. *Moraxella catarrhalis* have virulence factors such as β-lactamases, biofilm formation, MID/Hag, which mediates hemagglutination and non-immune binding of IgD, and a number of outer-membrane proteins involved in adherence to epithelial cells [232]. Thirty-one *Moraxella* isolates were identified in our survey (Figure 4): 51.6% *M. lucunata*, 22.6%; *M. catarrhalis*, 12.9%; *M. nonliquefaciens*; one *M. osloenis*; and three unspeciated *Moraxella*. Although there are reports of increasing frequency of *Moraxella* isolates in ocular infections [228,233], no increase in the number of isolates with time was observed in our survey. Interestingly, there were no isolates of *M. catarrhalis* collected after 2014, and all isolates of *M. nonliquefactiens*, *M. osloensis*, and unspeciated *Moraxella* were collected after 2018.

### 4.9. Pseudomonas

*P. aeruginosa* are ubiquitous rod-shape bacteria which, due to their simple nutritional needs and innate resistance to antibiotics and antiseptics, have been isolated from soil, water, human gastrointestinal tracts, sinks, showers, distilled water [234] and are commonly isolated from uninfected eyes (Table 1). *Pseudomonas* sp. have also been identified by 16s rDNA as part of the conjunctival microbiota [154,155]. *P. aeruginosa* are causes of acute conjunctivitis [4,235], dacryocystitis [84], post-surgical and post-traumatic endophthalmitis [236,237], endogenous endophthalmitis [238], and are the major cause of contact lens-associated keratitis [239,240,241]. Recently, deaths have been reported following *P. aeruginosa* ocular infections resulting from contaminated eye drops [242]. *P. aeruginosa* may sequester in niches in ocular glands during keratitis, potentially leading to spread to other extraocular sites [243].

Keratitis infections of contact lens wears are associated with the phylogenetic subgroup encoding the cytotoxin exotoxin U gene (*ExoU*) [241,244], while keratitis infections of populations with lower contact lens use are predominantly caused by species with genes encoding exotoxin S (*ExoS*) [2]. Enzymes such as elastase B, protease IV, and *P. aeruginosa* small protease [245,246,247] have been reported to play a role in experimental *Pseudomonas* keratitis. Although *P. aeruginosa* are the most commonly isolated pseudomonads, other species have been recovered from ocular infections, such as and *P. putida* from a case of conjunctivitis [248] and *P. fluorescens* from a case of endophthalmitis [249].

The role of the exotoxins mentioned above in *P. aeruginosa* keratitis have been widely studied, as has the pathogenic profile of cytotoxic and invasive strains and the intracellular nature of *P. aeruginosa* in the corneal epithelium. *Pseudomonas* contact lens-associated keratitis is the result of the confluence of the widespread use of ocular prosthetic devices and an opportunistic pathogen with a large and versatile arsenal of virulence factors [250]. The healthy, undamaged cornea is naturally resistant to *Pseudomonas* infection [251]. A full description of the many virulence factors involved in overcoming the innate resistance of the healthy cornea to infection is beyond the scope of this review but has been ably studied and reviewed by Fleisizg et al. [252]. To our knowledge, investigation into the specific virulence factors involved in the development and pathogenesis of *Pseudomonas* endophthalmitis has not been undertaken.

Multidrug resistance to antibiotics is common among *P. aeruginosa* due to chromosomally encoded genes and the ability to acquire mobile genetic elements [253]. Because of this, *P. aeruginosa* are members of the ESKAPE group of bacterial pathogens [66]. The most effective antibiotics against *P. aeruginosa* keratitis have been reported to be levofloxacin [92,253,254], ciprofloxacin [92,253], and amikacin [253], with reported sensitivities of 94.6%, 90.9%, and 90.2%, respectively [253]. However, resistance patterns vary from country to country [253]. In the ARMOR study noted above, all ocular isolates of *P. aeruginosa* were sensitive to all tested antibiotics, with infrequent in vitro resistance to polymyxin B (8.6%), tobramycin (2.5%), and fluoroquinolones (5.2–7.4%) [119]. In our survey, we report 90 isolates of *P. aeruginosa:* 64.4% from the cornea, 12.22% from the conjunctiva, and the remainder from other ocular sites (Figure 3 and Figure 4).

### 4.10. Proteus mirabilis

*Proteus mirabilis* (*P. mirabilis*) are motile, rod-shaped ubiquitous bacteria found in soil and water and are commensal inhabitants of animal gastrointestinal tracts [255]. *P. mirabilis* possesses a number of virulence factors, such as motility, proteases, and hemolysins, *Proteus* has a toxic agglutinin which promotes autoaggregation of bacteria and lysis of bladder cells in vitro, and a ZapA metalloprotease which cleaves IgA, IgG, complement proteins C1q and C3, and proteins such as fibronectin, actin, and collagen [255]. *P. mirabilis* can cause infections of the gastrointestinal tract and wounds but is most commonly known for catheter-associated urinary tract infections [255]. *P. mirabilis* is a rare cause of keratitis [256] and endophthalmitis [257]. Mo et al. [256], in their retrospective review of 26 culture-proven cases of *P. mirabilis* keratitis, reported that all isolates were susceptible to ciprofloxacin, ofloxacin, moxifloxacin, gatifloxacin, and cefazolin. Although capable of causing serious disease, *P. mirabilis* keratitis has been reported to respond well to prompt and appropriate treatment. Our survey reports four *P. mirabilis* isolates, three isolated from the cornea and one isolated from the lacrimal gland.

### 4.11. Serratia

*Serratia* is a motile, rod-shaped anaerobe widely found in soil, plants, and water [258,259]. The most common species isolated from infections is *Serratia marcescens* (*S. marcescens*). Isolation of *Serratia liquefactions* (*S. liquefaciens*) is less commonly reported [260]. *S. marcescens* virulence factors include motility, fimbriae for adherence, several hemolysins which are toxic to different cell types, metalloproteinase, gelatinase, endonuclease, and proteases [260,261,262]. In a mouse model of keratitis, *S. marcescens* induced corneal inflammation by activating TLR4 and TLR5, inducing CXC chemokine production, which recruited neutrophils to the corneal stroma, resulting in corneal thickening and opacity [263]. Using a rabbit model of keratitis, Romanowski et al. [264] demonstrated that the *Serratia* Rcs stress response system is regulated by *GumB*. Mutation of *GumB* resulted in a greater than 50-fold reduction in *S. marcescens* proliferation and a reduction in inflammatory markers, indicating that *GumB* is a key mediator of *S. marcescens* corneal virulence [264].

*Serratia* species are inherently resistant to a wide range of antibiotics, including ampicillin, amoxicillin, and colistin [259]. *S. marcescens* frequently has both chromosomal and plasmid-mediated resistance to multiple antibiotics [260]. *S. marcescens* cause nosocomial infections ranging from pneumonia and endocarditis to urinary tract infections [265]. In the eye, *S. marcescens* has been reported to cause keratitis, lacrimal duct infection, endophthalmitis, and conjunctivitis [266]. *S. liquefaciens* has been reported in a case of keratitis [267]. *S. marcescens* keratitis is associated with the presence of an abnormal corneal surface, the use of topical medications, and contact lens use [266,268]. In their retrospective study of 24 cases of *S. marcescens* keratitis, Mah-Sadorra et al. [268] reported that a good clinical response was obtained with topical fluoroquinolones and fortified aminoglycoside drops. In their review of 51 patients, Atta et al. [266] reported that most patients with *Serratia* keratitis responded well to antibiotic drops and rarely required adjunctive treatment or surgical interventions. *Serratia* has also been reported to cause endogenous endophthalmitis resulting in visual impairment and loss of vision [269,270]. In our survey, 10 isolates of *Serratia* are reported: nine *S. marcescens* (five from the cornea, two from the canaliculus, one from the vitreous, and one from the anterior chamber), and one *S. liquefaciens* isolate from the cornea.

### 4.12. Stenotrophomonas maltophilia

*Stenotrophomonas maltophilia* (*S. maltophilia*) is a ubiquitous environmental rod-shaped bacterium with intrinsic antibiotic resistance to β-lactams and aminoglycosides. *Stenotrophomonas* is capable of causing a variety of nosocomial and community-acquired infections, primarily in immunocompromised patient populations [271]. *S. maltophilia* is frequently isolated from hospitalized pneumonia patients but also causes infections of the respiratory tract, central nervous system, gastrointestinal tract, urinary tract, soft tissues, and bone [271]. The virulence factors of *S. maltophilia* include motility, biofilm formation, and production of extracellular enzymes, such as DNase, proteases, lipases, hyaluronidase, and hemolysin [271]. *S. maltophilia* is a rare cause of ocular infections but has been reported as a cause of cellulitis, conjunctivitis, dacryocystitis, endophthalmitis, and keratitis [272,273,274,275]. In pediatric keratitis cases, *S. maltophilia* was the second most commonly identified species after *P. aeruginosa* [240]. In their retrospective review of 16 culture-proven cases of *S. maltophilia* keratitis, Park et al. [275] reported that most cases were associated with ocular surface instability, such as trauma or contact lens use, and that the treatment of choice was a mixed use of a fluoroquinolone, beta-lactam, and aminoglycoside. Our survey reports eight samples of *S. maltophilia* isolated from the cornea, contact lenses, and conjunctiva.

## 5. Hapax Legomenon Isolates

### 5.1. Abiotrophia, Actinomyces, and Brevundimonas diminuta

Eleven genera were only isolated once in our survey (Figure 2). Three of these, *Abiotrophia* sp. (Gram-positive cocci, corneal isolate), *Actinomyces* sp. (Gram-positive filamentous, corneal isolate), and *Brevundimonas diminuta* (Gram-negative bacillus, corneal isolate) have been reported as rare causes of ocular infections [276,277,278]. The isolates of *Abiotrophia*, *Actinomyces*, and *Brevundimonas diminuta* in our survey were all corneal isolates.

### 5.2. CDC Group EO-3, Cronobacter, and Lactobacillus

CDC Group EO-3 (Gram-negative coccobacillus, corneal isolate) has a single case report of peritonitis [279] in the literature, and to our knowledge, no reports of causing ocular infections. *Cronobacter sakazakii* (Gram-negative bacillus, unknown ocular location) is a common contaminant of dry plant-based foods and has caused foodborne outbreaks of necrotizing enterocolitis, septicemia, and meningitis among neonates [280]. The most recent US outbreak of *Cronobacter* infections was linked to contaminated infant formula powder [281]. To our knowledge, there are no literature reports of ocular infections due to *Cronobacter*. *Lactobacillus* sp. (Gram-positive bacillus from an unlisted ocular location) is widely distributed among plants and animals [282], is part of the ocular microbiota [254], and is not considered pathogenic [282]. However, *Lactobacillus* sp. is a rare cause of bacteremia in immunocompromised patients [283]. To our knowledge, there have been no reports of ocular infections caused by *Lactobacillus*, although there have been reports of the use of *Lactobacillus* in probiotic preparations to stimulate ocular immunity [284,285].

### 5.3. Neisseria

*Neisseria* sp. (Gram-negative diplococcus) is a cause of mucopurulent conjunctivitis and keratitis [286]. *Neisseria meningitidis* (*N. meningitidis*) virulence factors include pili, opacity-associated proteins, lipooligosaccharides, and capsular polysaccharides [287]. There are 13 clinically significant serogroups of *Neisseria* based on their capsular polysaccharides, and each has a specific geographic distribution [287]. In their study of 60 isolates of *Neisseria* keratitis, Kate et al. [286] reported that forty percent of isolates were only identified to the genus, 21.7% were caused by *N. elongata*, 16.7% by *N. meningitis*, 6.6% by *N. weaver*, and 5% by *N. mucosa*. Keratitis was often preceded by conjunctivitis, but keratitis also developed following a compromised ocular surface or ocular injury, particularly when topical corticosteroids were in use [286]. Keratitis normally responded well to topical antibiotics, and 100% of *Neisseria* keratitis isolates were susceptible to gatifloxacin, 94% susceptible to amikacin, 96% susceptible to chloramphenicol, and 96% susceptible to gentamycin [286].

Although a common cause of sexually transmitted diseases, *N. gonorrhoeae* is also a rare cause of ocular disease. Virulence factors include Type IV pili, lipooligosaccharides, porin, opacity proteins, and efflux pumps [288]. In their five-year retrospective review, Butler et al. [289] reported 15 cases of ocular gonococcal infections. The most common presenting features reported were a purulent discharge (93% of cases), hemorrhagic conjunctivitis (67% of cases), and pre-sepal cellulitis (60% of cases). All patients were treated with systemic antibiotics and topical chloramphenicol or ofloxacin. Corneal involvement was reported in 33% of cases, but there was no “significant” corneal melting or perforation [289]. Because corneal ulceration can rapidly progress to corneal melting and perforation, treatment must be prompt and effective [290,291]. Because of the increasing rates of *N. gonorrhoeae* resistance to penicillin, tetracycline, and fluoroquinolones, the Center for Disease Control recommends an intramuscular injection of ceftriaxone in a single dose with topical saline lavage of the eye and sexual partners of the patient should be referred for evaluation and treatment [292]. The single isolate of unspeciated *Neisseria* in our survey was isolated from the conjunctiva.

### 5.4. Pasteurella

*Pasteurella* sp. is a common Gram-negative coccobacilli resident of the oral and digestive tract of many birds and mammals, particularly dogs and cats [293]. *Pasteurella* is not part of normal human flora [294]. *Pasteurella* infections of soft tissues, bones, and wounds can result from animal bites and scratches [295]. Ocular infections with *Pasteurella* are rare, but bites and scratches to the eye can result in keratitis, conjunctivitis, or endophthalmitis, primarily from species *P. canis* and *P. multocida* [293,294]. Studies of the antibiotic susceptibilities of non-ocular clinical isolates have shown that most human isolates of *Pasteurella* are susceptible to moxifloxacin, amoxicillin, azithromycin, and clarithromycin [295]. Shah et al. [294] recommended fortified vancomycin and tobramycin for the treatment of *Pasteurella* keratitis. The single unspeciated *Pasteurella* isolate in our survey was a corneal isolate.

### 5.5. Providencia

*Providencia* sp. are Gram-negative motile rods that have been isolated from water and soil and are most commonly known for causing urinary tract and wound infections [296]. Human isolates of *Providencia* have been recovered from the axilla, blood, perineum, stool, urine, and wounds [297]. Risk factors for ocular infections with *Providencia* are immunocompromise and urinary tract infections, particularly among those with long-term indwelling urinary catheters [296]. *Providencia* form biofilms, have intrinsic resistance to colistin and tigecycline [298], may produce β-lactamase, and resistance to fluoroquinolones is on the increase [296]. *Providencia alcalifaciens* has been isolated from a case of keratitis [299], and *P. rettgeri* has been reported to cause keratitis, dacryocystitis, conjunctivitis, and endophthalmitis [296]. The single *Providencia* isolate in our survey was isolated from a cornea and was not speciated.

### 5.6. Rhodococcus

*Rhodococcus* sp. are Gram-positive to Gram-variable actinomycetes, are ubiquitous in soil and water, and are most commonly known for causing opportunistic infections of immunocompromised patients, particularly those with HIV [300,301]. The virulence factors of *Rhodococcus equi* include polysaccharide capsules, hemolytic enzymes, β-lactamases, and the ability to multiply inside macrophages [302]. *Rhodococcus* has been detected by 16S rDNA PCR from normal human eyes in repeated samples, indicating that this genus is part of the conjunctival microbiota [303]. *R. rhodochrous* [300], *R. globerulus* [304], and *R. ruber* [305] are reported but uncommon causes of keratitis. *R. erythropodes* and *R. luteus* have been isolated from cases of post-surgical endophthalmitis [306]. *R. gordoniae* was identified as a cause of eyelid infections [307]. A reported case of *R. globerulus* keratitis was treated with fortified vancomycin, amikacin, and erythromycin [304], and a case of *R. ruber* keratitis was treated with 2% amikacin [305]. Both cases responded well to the treatments. The single unspeciated *Rhodococcus* in our survey was a corneal isolate.

### 5.7. Rothia

*Rothia* sp. are Gram-positive rods that are resident flora of the upper respiratory tract and are responsible for a variety of infections, such as bacteremia, endocarditis, meningitis, and pneumonia, most commonly in immunocompromised patients [308,309]. *Rothia dentocariosa* and *R. mucilaginosa* are rare causes of endophthalmitis [310], and *R. dentocariosa* is a rare cause of keratitis [311,312]. Keratitis caused by *R. dentocariosa* has been treated successfully with cefuroxime and penicillin [312], and the case reported by Morley and Tuft [311] was sensitive to ofloxacin, chlorophenol, and cefuroxime. The single unspeciated *Rothia* isolate in our survey was a corneal isolate.

## 6. Conclusions

Diagnosing the cause of any infection can be a challenging task. Once the culture results return days or weeks later, the question is whether the bacteria in the report is the cause of the infection. As will have been noted by the reader, many of the bacteria listed in this review are commensal organisms and can be easily isolated from healthy individuals. Complicating the matter is the fact that in keratitis samples, only 40–56% of cultures are positive for growth [313,314], and with endophthalmitis cultures, only 40–70% of cultures are positive for growth [315]. Essential to diagnosing and properly treating the cause of infection is understanding the factors involved, such as which pathogens in that area cause that type of infection, whether multidrug resistance is prevalent in that region, and the immunocompetent state of the patient [315].

Efforts to shorten the diagnostic time for identifying possible pathogens in eye infections by using PCR, whole genome sequencing (WGS), and Nanopore Sequencing [315] are important. In a study of samples from cases of endophthalmitis, WGS was shown to be more sensitive than culturing or 16S sequencing for the identification of pathogens [315]. Methods such as WGS have the potential to detect all organisms (bacterial, fungal, and viral) in environmental samples, which could improve diagnostics and treatment of complex polymicrobial infections. WGS also allows for the analysis of genomes for virulence factors and antibiotic resistance genes [315], which could inform the treating physician about the potential severity of the infection as it evolves. However, as most eye care professionals know, the majority of keratitis and endophthalmitis patients respond very well to empirical treatments, and culture results do not often change the treatment regimen [316]. In one survey, only 35.1% of corneal ulcers were actually cultured [317]. While this may be a cause for concern, it also demonstrates that we have a good understanding of what pathogens are likely causing an infection and that our treatments are currently effective for the majority of cases. Also of concern is complacency in assuming that current treatment regimens will be successful for all ocular infections. Continual monitoring of the resident flora of the eye and the organisms involved in eye infections is essential to identify emerging antibiotic resistance and virulence profiles. As reports of high-throughput sequencing of the ocular microbiota are becoming commonplace, it is important to note that the organisms reported in our survey only covered culturable bacteria. While novel strategies such as rapid Nanopore Sequencing may identify previously undetected organisms [315], this will not circumvent the need to culture bacteria. In order to track antibiotic resistance or to understand the function of genomic data, it is necessary to have an organism to test and assays to determine the extent to which expression of resistance or virulence genes occurs. Pathogens are constantly evolving to adapt to antibiotic treatments, new pathogens are emerging to exploit vulnerable human populations, and some of these vulnerable populations are growing in number.

In this review, we report a survey of the spectrum of bacteria recovered from ocular infections and banked over the course of 10 years. A limitation of this survey is the absence of correlative patient data. This review is by no means an exhaustive summary of all the possible bacteria that can be cultured from human eyes, but it illustrates the variety of species that have been isolated from a population at a single institute over a specific period of time. The phrase “ocular infections” covers a wide scope of diseases, ranging from conjunctivitis to pan-ophthalmitis. While certain ocular infections, such as keratitis and endophthalmitis, have been relatively well studied, that is true only in regard to the most common pathogens which cause the disease. As this review has shown, there is a vast spectrum of bacteria capable of colonizing the ocular surface, and only a few have been studied well enough that treatments have moved beyond “empirical”.

Several studies have sampled the ocular flora of healthy eyes and have shown that the predominant ocular bacteria in healthy conjunctiva are composed of CoNS and Gram-positive cocci (Table 1). In studies of keratitis isolates, Gram-positive cocci and CoNS are also the most common type of bacteria isolated (Table 4). We found the same trend of Gram-positive cocci and CoNS being the most common type of bacteria isolated (Figure 2). Table 1 and Table 4 summarize the results of a number of surveys of ocular bacterial isolates from healthy eyes and isolates from keratitis cases, respectively. Comparisons of our data with the bacteria isolated from healthy and keratitis eyes are difficult because of the large range of numbers in those studies, but some general comparisons can be made. In our survey, 79.51% of the bacteria isolated were Gram-positive bacteria, which is in the upper range of both groups of isolates. However, the 34.1% CoNS and 15.28% *S. aureus* in our survey are in the lower bounds of both groups, indicating that this population has higher numbers of Gram-positive cocci other than *Staphylococcus*. Our survey contained 20.38% Gram-negative bacteria, which is in the upper range reported in keratitis eyes and the lower range reported in healthy eyes. These data suggest that the species of bacteria found in our sample of isolates from patients visiting an eye care facility might not be representative of a healthy population.

Studies of bacterial infections tend to focus on single species, although it is well-known that bacteria in the natural environment exist as part of a community of organisms. Delbeke et al. [318], in their systematic review of 11 ocular microbiome studies, define the core ocular microbiome as being composed of *Corynebacterium*, *Acinetobacter*, *Staphylococcus*, *Propionibacterium*, and *Streptococcus*. All of these genera have been reviewed above as having pathogenic potential. Prolonged contact lens wear and repeated intravitreal injections have provided normally symbiotic bacteria an avenue to ocular pathogens by altering the epithelial surface of the eye or by breaching the protective layers of the eye. The study by Shin et al. [155] comparing the ocular microbiome of contact lens wears with noncontact lens wear showed that contact lens use was associated with a decrease in the relative abundance of *Corynebacterium*, *Staphylococcus*, and *Propionibacterium* and an increase in the relative abundance of *Pseudomonas* and *Acinetobacter*. Understanding the bacterial communities that populate the ocular surface enables us to prevent the unintended enrichment of bacteria with pathogenic potential into the eye and provides us with information as to the likely pathogens when infections do occur.

Ocular infections of any kind are not trivial inconveniences to those who suffer from them. Even a self-limiting case of bacterial conjunctivitis can result in lost wages, time lost from school, parental time away from work, and the social stigma of an unsightly contagious ocular infection. Because of the delicate nature and great importance of ocular tissues, medical care must be prompt and effective. Because of the continual adaptation of known pathogens and the continual emergence of new pathogens, it is vital that the topic of ocular flora continue to be studied.

## Figures and Tables

**Table 2:** Primer sequences for amplification of virulence genes in PCR of ocular bacterial isolates.

	Gene		Sequences (5′-3′)
**Clumping factor [10]**	*clf*	Fwd	CGA TTG GCG TGG CTT CAG
		Rev	GCC AGT AGC CAA TGT CAC
**Fibronectin-binding protein B [10]**	*fnbA*	Fwd	GCG GAG ATC AAA GAC AA
		Rev	CCA TCT ATA GCT GTG TGG
**Fibronectin-binding protein A [10]**	*fnbB*	Fwd	GGA GAA GGA ATT AAG GCG
		Rev	GCC GTC GCC TTG AGC GT
**Gamma-hemolysin A, B. and C [107]**	*hlg*	Fwd	GTC AYA GAG TCC ATA ATG CA TTT AA
		Rev	CAC CAA ATG TAT AGC CTA AAG TG
**Alpha-hemolysin [10]**	*hla*	Fwd	GGT TTA GCC TGG CCT TC
		Rev	CAT CAC GAA CTC GTT CG
**Beta-hemolysin [10]**	*hlb*	Fwd	GCC AAA GCC GAA TCT AAG
		Rev	CGC ATA TAC ATC CCA TGG C
**LukE-LukD [107]**	*lukE-lukD*	Fwd	TGAAAAAGGTTCAAAGTTGATACGAG
		Rev	TGTATTCGATAGCAAAAGCAGTGCA
**Mec A [10]**	*mecA*	Fwd	GTA GAA ATG ACT GAA CGT CCG ATA A
		Rev	CCA ATT CCA CAT TGT TTC GGT CTA A
**PVL [107]**	*lukS-PV-lukF-PV*	Fwd	ATC ATT AGG TAA AAA TGT CTG GAC ATGATC CA
		Rev	GCATCAASTGTATTGGATAGCAAAAGC
**TSST-1 [10]**	*tst*	Fwd	AAG CCC TTT GTT GCT TGC G
		Rev	ATC GAA CTT TGG CCC ATA CTT T

**Table 3:** Virulence genes of MRSA and MSSA *S. aureus* ocular isolates, as determined by PCR. The number of isolates is designated in parentheses. Gene identities can be found in Table 2.

	Gene									
	clf	fnbA	fnbB	hla	hlb	mecA	tst	hlg	luk	PVL
MRSA (48)	100.0%	95.8%	77.1%	85.4%	75.0%	100.0%	0.0%	10.4%	85.4%	37.5%
MSSA (81)	97.5%	97.5%	58.0%	80.2%	79.0%	0.0%	6.2%	24.7%	71.6%	14.8%
All (129)	98.4%	96.9%	65.1%	82.2%	77.5%	37.2%	3.9%	19.4%	76.7%	23.3%

**Table 4:** Species isolated from keratitis cases in various studies across the world.

	Period of Study	Location	Gram-Positive	*Bacillus*	CoNS	*S. aureus*	*S. epidermidis*	Streptoccoci	*S. pneumoniae*	Gram-negative	*Moraxella*	*Pseudomonas* sp.	*P. aeruginosa*	*Serratia*
**Alexandrakis [126]**	1990–1998	USA, Florida	48.0%			19.4%	1.3%	6.7%		49.6%			25.7%	7.6%
**Schaefer [127]**	1997–1998	Switzerland		1%		22%	40%	5%	8%		5%	9%		5%
**Leck [128]**	1999–2001	India, Tamil Nadu		0.9%	24.7%	2.1%		46.80%	26.4%			14.9%	14%	
**Leck [128]**	1999–2001	Ghana		0.0%	5.0%	5.0%		20.0%	15.0%			52.5%		
**Lam [129]**	1997–1998	Hong Kong	46.8%			11.4%				53.2%			6.3%	
**Bourcier [82]**	1998–1999	France, Paris	83.1%		48.3%	7.7%		9.2%	3.4%	16.9%	0.5%		10.1%	5.3%
**Zhang [130]**	2001–2002	China, Beijing	67.62%	2.16%		6%	15.83%		7.91%	32.38%		17.99%		0.71%
	2003–2004	China, Beijing	59.28%	0.71%		8%	12.14%		7.14%	40.72%		22.15%		2.14%
**Geethakumari [131]**	2007–2009	India, Kerala			9.09%	15.9%			26.14%			26.14%		
**Orlans [132]**	1999–2004	UK, Oxford	56.1%	1%	20.1%	18.7%			3.6%	43.9%	3.6%	25.9%	20.9%	3.5%
	2004–2009	UK, Oxford	52.4%	0%	32.0%	9.4%			2.3%	47.7%	6.2%	31.20%	28.1%	0%
**Lichtinger [6]**	2000–2003	Canada, Toronto	81.4%		40.3%	20.0%		16.3%		19%	3%		7.2%	3.2%
	2004–2007	Canada, Toronto	74%		33.7%	5.7%		20.0%		26%	5.1%		15.6%	3.8%
	2008–2010	Canada Toronto	69%		33.1%	16.9%		15.2%		31%	8.4%		21.3%	2.8%
**Tan [133]**	2004–2006	UK, Manchester	71.9%	4.4%	35.0%	14.5%		12.6%		28.1%	4.4%	12.6%		3.6%
	2007–2009	UK, Manchester	72.7%	5.6%	30.6%	15.9%		12.9%		27.3%	5.6%	9.4%		2.3%
	2010–2012	UK, Manchester	68.0%	1.9%	23.9%	15.6%		17.9%		32.3%	4.8%	12.9%		3.5%
	2013–2015	UK, Manchester	66.2%	1.7%	15.4%	21.7%		16.1%		36.1%	13.0%	10.0%		3.3%
**Lin [134]**	2006–2007	India, Southeast							37.0%			21.6%		
	2007–2008	India, Southeast							32.5%			27.0%		
	2008–2009	India, Southeast							35.5%			24.5%		
**Ting [135]**	2008–2012	UK, England	65.8%	5.8%	21.1%	17.4%		16.8%		34.2%	10.0%	16.3%		
	2013–2017	UK, England	74.7%	8.6%	34.3%	13.1%		10.6%		25.3%	10.6%	7.8%		
**Al-Dhahari [9]**	2011–2014	Saudi Arabia	91.4%		61.4%	11.6%	47.2%		7.8%		2.2%		6%	
**Hsiao [136]**	2003–2007	Taiwan	46.2%	8.3%	15.50%	9.40%			4.4%	53.8%			26.1%	5.7%
	2008–2012	Taiwan	54.8%	7.4%	16.40%	7.60%			1.7%	45.2%			22.9%	4.8%
**Gautam [137]**	2017–2018	Nepal				21%	56.00%		2.0%				12.0%	
**Sagerfors [35]**	2004–2014	Sweden	79.1%		38.0%	14.5%			3.0%	20.9%	7%		6.4%	

## Data Availability

The data presented in this study are available on request from the corresponding author.
